# The development and feasibility of a randomised family-based physical activity promotion intervention: the Families Reporting Every Step to Health (FRESH) study

**DOI:** 10.1186/s40814-019-0408-7

**Published:** 2019-02-09

**Authors:** Justin M. Guagliano, Helen Elizabeth Brown, Emma Coombes, Claire Hughes, Andy P. Jones, Katie L. Morton, Edward C. F. Wilson, Esther M. F. van Sluijs

**Affiliations:** 1 MRC Epidemiology Unit and UKCRC Centre for Diet and Activity Research, University of Cambridge, Cambridge, UK; 2 Norwich Medical School and UKCRC Centre for Diet and Activity Research, University of East Anglia, Norwich, UK; 3 Centre for Family Research, University of Cambridge, Cambridge, UK; 4 Cambridge Centre for Health Services Research, Institute of Public Health, University of Cambridge, Cambridge, UK

**Keywords:** Children, Youth, Parent, Mothers, Fathers, Mums, Dads, Co-participation, Co-physical activity

## Abstract

**Background:**

There is a need for high-quality research aiming to increase physical activity in families. This study assessed the feasibility and acceptability of FRESH (Families Reporting Every Step to Health), a child-led family-based physical activity intervention delivered online.

**Methods:**

In a two-armed randomised feasibility study, 12 families (with an 8–10-year-old index child) were allocated to a ‘child-only’ (CO) or ‘family’ arm (FAM) of the theory-based FRESH intervention. Both received access to the FRESH website, allowing participants to select step challenges to ‘travel’ to target cities around the world, log their steps, and track their progress as they virtually globetrot. Only index children wore pedometers in CO; in FAM, all family members wore pedometers and worked towards collective goals. All family members were eligible to participate in the evaluation. Mixed-methods process evaluation (questionnaires and family focus groups) at 6-week follow-up consisted of completing questionnaires assessing acceptability of the intervention and accompanying effectiveness evaluation, focussed on physical (e.g. fitness, blood pressure), psychosocial (e.g. social support), and behavioural (e.g. objectively-measured family physical activity) measures.

**Results:**

All families were retained (32 participants). Parents enjoyed FRESH and all children found it fun. More FAM children wanted to continue with FRESH, found the website easy to use, and enjoyed wearing pedometers. FAM children also found it easier to reach goals. Most CO families would have preferred whole family participation. Compared to CO, FAM exhibited greater website engagement as they travelled to more cities (36 ± 11 vs. 13 ± 8) and failed fewer challenges (1.5 ± 1 vs. 3 ± 1). Focus groups also revealed that most families wanted elements of competition. All children enjoyed being part of the evaluation, and adults disagreed that there were too many intervention measures (overall, 2.4 ± 1.3) or that data collection took too long (overall, 2.2 ± 1.1).

**Conclusion:**

FRESH was feasible and acceptable to participating families; however, findings favoured the FAM group. Recruitment, intervention fidelity and delivery and some measurement procedures are particular areas that require further attention for optimisation. Testing the preliminary effectiveness of FRESH on family physical activity is a necessary next step.

**Trial registration:**

This study was registered and given an International Standard Randomised Controlled Trials Number (ISRCTN12789422). Registered 16 March 2016. http://www.isrctn.com/ISRCTN12789422

## Introduction

Recent systematic reviews confirm numerous health benefits of regular physical activity for children [1, 2]. Nevertheless, approximately 80% of children in the UK do not meet the recommended 60 min of moderate-to-vigorous intensity physical activity (MVPA) every day [3]. Additionally, as children become less active in adolescence [4], there is a need for physical activity promotion [5, 6]. Observational data indicates that children are less active after school and at weekends, compared to while at school [7–9]. To date, however, physical activity promotion efforts have been conducted predominantly in the school setting, despite the impact of these school-based interventions on overall physical activity being questioned [10]. Family-based physical activity interventions may therefore present a promising avenue to promote children’s activity [11].

Previous evidence indicates that home-based physical activity interventions are potentially more effective than those requiring the family to travel to community or other intervention locations [12, 13]. Further, it is unlikely that a change in children’s physical activity levels will be sustained long-term without the involvement of wider family members [14–16]. Many studies, however, only focus on promoting child physical activity instead of considering the family as a unit that may work together to change behaviour [17].

Calls for physical activity research in young people and families highlight the dearth of research in this area [18] and the need to develop and evaluate innovative interventions targeting children and families. Responding to this challenge, in this paper, we (1) describe the development of the Families Reporting Every Step to Health (FRESH) intervention and recruitment strategy; (2) assess the feasibility and acceptability of the FRESH recruitment strategy, intervention (including intervention fidelity), and accompanying outcome evaluation; and (3) explore how FRESH could be optimised through a mixed-methods process evaluation.

## Methods

Reporting of this study was guided by the Consolidated Standards of Reporting Trials extension to randomised pilot and feasibility trials guidelines [19] and the Template for Intervention Description and Replication [20]. This feasibility study received ethical approval from the Ethics Committee for the School of the Humanities and Social Sciences at the University of Cambridge and was prospectively registered (ISRCTN12789422).

### Study design — overview

FRESH was a 6-week two-armed, parallel-group, randomised feasibility study, using a 1:1 allocation ratio, aiming to recruit 20 families with an index child aged 8–10 years. Following baseline assessment, families were randomly allocated to one of two intervention arms. In the ‘child-only’ arm, the index child was the focus of the intervention, with family members simply providing support. In contrast, in the ‘family’ arm, all participating family members received the FRESH intervention (described later).

An independent statistician performed the randomisation procedure in Stata (version 14; StataCorp. TX: StataCorp LP) using a computer-generated algorithm and a randomised block design (blocks of 4) to ensure equal numbers in each condition.

### Participants

Families were eligible to participate when at least one child aged 8–10 years (hereafter referred to as index children) and at least one adult responsible for their care and living in their main household provided consent. Participants also needed to be able to partake in light-intensity physical activity (e.g. walking), have access to the Internet, and have a sufficient understanding of the English language. No restrictions were placed on family type (e.g. single parent, inclusion of grandparents, siblings). All other family members living in the index child’s main household were invited to participate but their participation was not required. Additionally, intervention and evaluation participation were separate; family members could take part in the intervention irrespective of participation in the accompanying evaluation and vice versa. Specific exclusion criteria only applied to the evaluation of this study and are outlined below.

### Setting

Families were recruited from rural Norfolk, a county in East Anglia, UK. Norfolk is 2074 mile^2^ and has a total estimated population of 898,400 [21], about half of which live rurally [22], where rural-urban disparities in physical activity have been previously reported [9, 23]. In accordance with the Office for National Statistics [24] classification, ‘rural’ was defined as having a postcode falling in a small town, village, hamlet, or dispersed settlement.

### Recruitment

#### Formative work informing the development of the FRESH recruitment strategy

The recruitment of families is known to be particularly challenging, and there is little evidence to suggest how best to engage families in physical activity research [14, 25]. To inform recruitment and retention, focus groups were conducted with 17 families (82 participants, consisting of 2–6 family members) [26]. Findings suggested (1) using a multi-faceted recruitment strategy and (2) highlighting the wide range of benefits of research participation (particularly social, health, and educational outcomes). These lessons explicitly contributed to the planned recruitment strategies for the current study, where we planned school- and community-based (e.g. Brownies/Cubs, community centres, GP practices) recruitment, highlighting the benefits of spending time together as a family in our recruitment material.

#### Recruitment protocol

To recruit schools and community-based organisations, we first contacted lead personnel (e.g. head teachers, physical education coordinators, heads of community-based organisations) by sending an information pack detailing the purpose of the study and all procedures, followed by a phone call if no response was obtained. Verbal or written approval was sought to send home study leaflets to children, circulate our leaflet to parents online, and send an online reminder to parents approximately 2 weeks later. In schools, we also sought permission to present to year 3–5 students at a scheduled assembly. Next, interested parents contacted the study team via e-mail or Freephone, after which eligibility was assessed and study information emailed. A baseline assessment appointment was then made with those families still interested in participating. Written informed consent was obtained for participating adults and written parental consent and child assent for each participating child prior to baseline assessments.

### Intervention selection and development

#### Reviewing the literature

We conducted a systematic review and meta-analysis and found a small, but significant, effect favouring the experimental groups of family-based interventions compared to controls (Cohen’s *d* = 0.41; 95% CI 0.15–0.67) [27]. This review highlighted the scarcity of family-based intervention studies that (1) clearly indicated intended behaviour change mechanisms, (2) employed objective measures of physical activity, (3) engaged with/assessed intervention effects on wider family members, and (4) were theory-based. The development of the FRESH intervention was then informed by a programme theory for family-based physical activity interventions [27]. This highlighted the value of (1) using goal-setting combined with reinforcement in the context of family constraints (e.g. lack of time or scheduling difficulties), (2) focussing on changing the family psychosocial environment (e.g. using the child as agent of change), and (3) focussing on something other than the health benefits of physical activity (e.g. time together as a family). These collective findings were considered when developing the FRESH intervention.

#### Intervention selection

Four potential intervention concepts were developed following initial work [26]. The four concepts were as follows: (1) *Buddy scheme*: Families would be paired or grouped to facilitate peer support for physical activity. (2) *Small changes*: Providing a resources toolkit to each family, containing information on making small changes to increase physical activity (e.g. active travel suggestions, such as getting off the bus a stop early). (3) *Sports equipment library***:** A ‘travelling library’ of a large range of sporting equipment would move through a community once a week allowing families to borrow equipment. (4) *Family challenge:* Families would be framed as a ‘team’, working towards a common goal (e.g. an overall step count to ‘walk around the world’).

These four concepts were then brought to families during a university-run community engagement event where children acted as researchers to identify which their family would enjoy most, and further refined during meetings with stakeholders (i.e. parents, teachers, family health practitioner). This led to the selection of an intervention that allowed families to work as a ‘team’, tracking their efforts towards a common goal, and receiving small rewards for progress (*family challenge* from above). This initial input from families and stakeholders was used as a starting point to develop FRESH in its current form.

#### FRESH intervention description and protocol

**Fig. 1:**
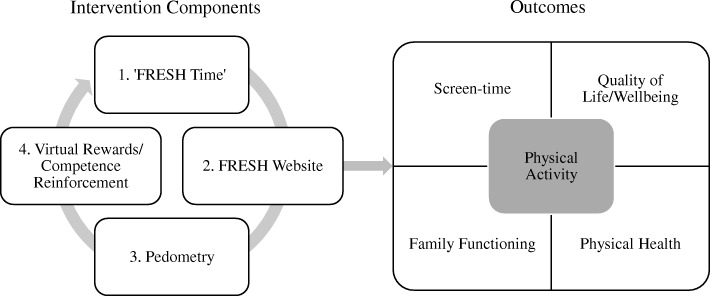
FRESH theoretical model

In brief, FRESH was primarily a goal-setting and self-monitoring intervention aimed at increasing physical activity in whole families. The Socio-Ecological Model (individual and interpersonal levels) [28] and Family Systems Theory [29] provided a framework for the intervention components. Within this framework, behaviour change strategies were guided by Self-Determination Theory [30]. A detailed description of the FRESH intervention components and associated behaviour change techniques, targeted Self-Determination Theory constructs, and hypothesised mediators are in Table 1. Additionally, the FRESH logic model can be found in Fig. 1.

**Table 1:** Summary of FRESH intervention components

Intervention components	Dose	Description	Behaviour change techniques	Targeted SDT constructs	Hypothesised mediators
1. Family time	Minimum 1×/week, 10–20 min	‘Family time’ provided an opportunity for index children^a^ and family members to plan PA, monitor their week’s steps, and discuss any potential PA barriers and strategies to overcome them by logging in their family action planners [27]. Regular family time was hypothesised to provide index children with: • A consistent (re)structured environment, where family members supported index children in choosing an optimally challenging and realistic goal (reflected as an easy, moderate, or difficult challenge on the FRESH website), breaking down goals into proximal (daily) and distal (weekly) step count targets, and providing praise and positive feedback on progress towards those goals. These strategies provide direct support for participants’ perceived competence [70]. • Consistent parental involvement which directly facilitates relatedness [70]. Parental involvement (via co-participation in PA) may also positively affect family connectedness [71]. • An opportunity for consistent autonomy support. Autonomy support has been shown to directly support participants’ autonomy and indirectly support their basic needs for competence and relatedness [72]. Additionally, index children were named their family’s team captain (i.e. change agent) where they were in charge of initiating ‘family time’. Evidence suggests that children may elicit changes to the psychosocial environment [27]; therefore, promoting the index children to the role of family ‘team captain’ may strengthen child buy-in, perceived autonomy, and improve intervention fidelity.	Goal-setting Self-monitoring Positive feedback on progress Social support Praise Positive reinforcement	Perceived competence Perceived relatedness Perceived autonomy	Family social norms for PA PA awareness Basic needs satisfaction PA motivation
2. FRESH website	Minimum 1×/week, 5–20 min	The FRESH website facilitated self-monitoring of step counts, and goal-setting through selecting challenges. Specifically, the FRESH website allowed families to choose one of three target cities to ‘walk to’ weekly, with the aim to eventually ‘walk’ around the world. Each week, families chose an easy, moderate, or difficult challenge, which represented a 0%, 5%, or 10% increase, respectively, relative to the average steps they had taken in preceding weeks. Increases were adjusted to 0%, 2.5%, and a 5% once adults and children accumulating an average of 10,000 and 12,000 steps/day, respectively. Families also had access to a general resources area with suggestions for activities that families could do together and a map for a visual representation of the locations families have travelled to.	Goal-setting Self-monitoring Positive feedback on progress Rewards	Perceived competence Perceived relatedness Perceived autonomy	Social support Family social norms for PA PA awareness Basic needs satisfaction PA motivation
3. Pedometry	Throughout intervention (6 weeks)	Participants were provided with pedometers for self-monitoring and immediate feedback. Pedometers are simple to use and convenient and are associated with effective interventions for increasing parent-child physical activity [73]. Index children logged their steps (and their family members’ steps) into the FRESH website and/or onto the family action planners, which allowed participants to view their progress towards their proximal and distal step goals.	Self-monitoring Immediate feedback	Perceived competence Perceived autonomy	Social support Family social norms for PA PA awareness Basic needs satisfaction PA motivation
4. Virtual rewards/competence reinforcement	~ 1×/week (6 weeks)	To praise effort (i.e. competence reinforcement), participants received supportive messages, virtual passport stamps (i.e. virtual rewards), and access reinforcement materials (i.e. interactive multimedia information about the cities they have visited) on the FRESH website as they completed challenges to various cities around the world. Participants received 2–4 passport stamps for completed challenges (i.e. as difficulty increased, more stamps were awarded) and 1 passport stamp for an incomplete challenge.	Feedback on progress Rewards	Perceived competence	Basic needs satisfaction PA awareness

^a^The index child refers to the child aged 8–10 years in the family. *PA* physical activity, *SDT* Self-Determination Theory

A week after baseline measures, a facilitator visited all families for a ‘kick-off’ meeting to introduce families to the intervention components and accompanying materials (e.g. family action planner). The main purpose of this meeting was to familiarise families with the website and prompt families to schedule regular ‘family time’ meetings (minimum 1/week) where they reviewed and updated their family action planner. All meetings occurred in participating families’ home and lasted approximately an hour. Participant-initiated distant support was available for the duration of the intervention.

A detailed description of the FRESH intervention components can be found in Table 1. At the start of each new weekly challenge, families had family time, where they selected a challenge on the FRESH website and filled in their action planners. The FRESH website allowed families to choose one of three target cities to ‘walk to’ each week with the aim to eventually ‘walk’ around the world. The FRESH website primarily facilitated the self-monitoring of step counts and goal-setting through selecting challenges of varying difficulty. In both study conditions, children were allocated the role of ‘team captain’, leading on destination selection and uploading steps online. Families were to wear their pedometers for as long as possible daily to capture their steps and asked to upload step counts at least once weekly. After completing a challenge, families received effort-praising messages and virtual rewards (i.e. virtual passport stamps), as well as track their progress around the world and access reinforcement materials on the FRESH website (i.e. interactive information about the cities they have walked past during their challenge). If a family did not complete a challenge, to praise their effort, they progressed to a hidden city along their challenge route and still received a supportive message, virtual passport stamp, and access reinforcement materials. Completing a challenge (or if the week ended) incited the next family time meeting, where the above cycle was repeated (see cycle in Fig. 1).

#### Refining the FRESH intervention

The FRESH intervention as initially developed was further developed through public involvement activities. We sought input from children (*n* = 7) through a talk-aloud session regarding the layout and design of the FRESH website and also from families (*n* = 2) who pilot-tested the intervention protocol described above. Overall, the FRESH intervention was well received, children found the website easy to navigate, and no changes were made to the protocol. However, based on participants’ suggestions, minor changes were made to the intervention website. For example, participants found it discouraging when they participated in activities that could not be captured by their pedometers (e.g. swimming). Therefore, we added a ‘step calculator’ to the website that enabled participants to estimate the number of steps various activities, such as swimming, would give them, using data from a readily available activity-to-step converter online [31].

#### Outcome evaluation measures

As part of this study, we aimed to assess the feasibility and acceptability of the planned outcome evaluation (i.e. not effectiveness); therefore, here we briefly describe the measures included to provide an overview of what the outcome evaluation entails. Table 2 outlines the measures taken, including assessment order and estimated duration. Data collection was carried out by two trained research staff in participating families’ homes. Outcomes were assessed at baseline (prior to randomisation) and follow-up (6 weeks) on all participating family members (excluding children ≤ 2 years). All consenting family members took part in measurements, irrespective of intervention allocation and participation.

**Table 2:** Order of measures and estimated duration of data collection for each time point^a^

Measure	Duration
1. Anthropometric measures (height, weight, waist circumference)	5 min/person
2. Questionnaires^b^	20 min/family
3. Blood pressure	10 min/person
4. Step test (aerobic fitness)	Prep: 5 min/person Test: 8 min/family
5. Accelerometer and GPS explanation	5 min/family
6. Fictional family holiday (family functioning)	10 min/family
Total length of measurements	Minimum of 73 min
Total length of visit (including consent process)	Minimum of 88 min

^a^Estimate based on a three-person household, total time increases by ~ 30 min per additional family member. ^b^Questionnaires completed during data collection included: a child or parent questionnaire (per person), a family out-of-pocket physical activity expenditure questionnaire (per family), and a child or adult process evaluation questionnaire (per person; follow-up only)

### Physical activity and location

To assess individual physical activity, and family co-participation in physical activity, participants were asked to simultaneously wear an ActiGraph GT3X+ tri-axial accelerometer (ActiGraph LLC; Pensacola, Florida) and QStarz Travel Recorder BT1000X global positioning system (GPS) monitor (QStarz; Taipei, Taiwan). Participants wore the monitors affixed at each hip on an elastic belt during waking hours for 7 consecutive days. A valid week was defined as ≥ 600 min/day from 3 weekdays and 1 weekend day over the 7-day measurement period [32]. Non-wear was defined as ≥ 90 min consecutive zeros using vector magnitude. ActiGraph accelerometers have been shown to be valid and reliable devices for the measurement of physical activity levels in children and adults [33–35]; the GPS monitor used has been shown to have high static and dynamic validity in a variety of settings [36].

Accelerometer and GPS data were matched using Java, after which data points that had a time difference of ≤ 30 s between the accelerometer timestamp and that of its matched GPS location were considered valid for inclusion. Matched data points with a time difference greater than this, for example where the GPS was switched off or had lost signal, were considered as missing locational information because the participant might have moved to a new unrecorded location. From the matched data, we computed minutes per day that the GPS had maintained a signal and was therefore recording the participants’ location, as an indicator of data completeness. Only wear time data will be presented in the current paper; therefore, we have only provided information relevant to estimating wear time using both monitors.

#### Health outcomes

Aerobic fitness was measured using an 8-min submaximal step test [37]. Children < 8 years were excluded from the aerobic fitness test. Height, weight, waist circumference, and blood pressure (OMRON 705IT) were measured according to standardised operating procedures. Body mass index was calculated and converted into age- and sex-specific percentiles using standard growth charts for children [38].

#### Behavioural and psychosocial measures

Questionnaires assessed behavioural and psychosocial measures: adult and child screen-time use [39–42]; quality of life [43–46]; family co-participation in physical activity [42]; physical activity awareness [47, 48]; family social norms for physical activity [49, 50]; family support [49]; children’s and adult’s motivation for physical activity [51, 52]; children’s perceived autonomy, competence, and relatedness [52]; and family functioning [53]. Children ≤ 4 years did not complete this questionnaire.

#### Family functioning

The Fictional Family Holiday paradigm, a 10-min video-recorded activity where families were asked to write out a week-long holiday itinerary with unlimited budget, was used to assess family functioning via family relationships [53] and connectedness [54]. This is because the activity requires ‘power sharing’ (i.e. taking turns) and prompts the viewpoints of all family members on the topic, eliciting both individuality (through suggestions for destinations/activities or disagreements) and connectedness (through agreements, questions, or initiating compromise) contributing to the family’s final plan [53].

#### Family out-of-pocket expenditure for physical activity

Family expenditure related to physical activity was collected via a questionnaire that was developed and tested for the current study and completed by one adult for their whole family. The questionnaire comprised two questions about expenditure related to membership fees and subscriptions (e.g. for sports clubs, fitness centres) and sports equipment (e.g. sportswear, gadgets).

### Process evaluation

A mixed-methods process evaluation was conducted at the end of the 6-week intervention. Adults self-reported their overall opinion of FRESH, their opinion of the intervention components and measurements, and suggestions for improvement using opened-ended and 5-point Likert-scale questions (1 = strongly disagree, 5 = strongly agree). Children also self-reported on the above topics, responding to dichotomous ‘yes/no’ questions. Semi-structured focus groups were also conducted with 11/12 families (1 family declined participation) focussing on families’ perceived acceptability of individual FRESH intervention components, intervention fidelity, challenges/barriers engaging with FRESH, and suggested improvements. The mean focus group duration was 34 ± 10 min (range = 17–50 min). All focus groups were audio-recorded and transcribed verbatim.

### Data analysis

#### Quantitative data

Frequencies, percentages, means, and standard deviations were calculated to describe data related to recruitment, retention, fidelity, intervention optimisation, website engagement, and outcome measures.

#### Qualitative data

Using a long table approach, a content analysis was conducted using existing guidelines [55]. Specifically, the analysis was conducted in two separate phases. During the data organisation phase, text from each transcript was divided into segments (meaning units) to produce a set of concepts that reflected meaningful pieces of information [55]. Tags were then assigned to each meaning unit. Tagging was performed by one researcher, with a second double-tagging ~ 25% of transcripts. For the data interpretation phase, the inventory of tags from all transcripts was examined by two researchers, which led to the emergence of themes and sub-themes within each overarching category.

## Results

### Findings related to recruitment and retention

Only school-based recruitment was employed due to intervention development delays. Of the 11 schools approached, 3 declined (too busy: *n* = 2; doing enough physical activity promotion already, *n* = 1) and 3 did not respond. Five schools with an estimated 437 eligible students agreed (reach).

**Fig. 2:**
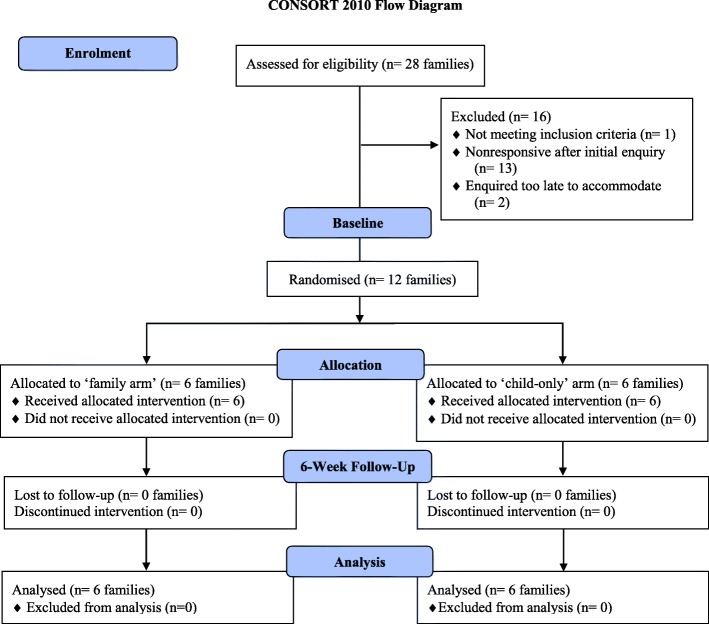
Participant flow diagram

Figure 2 shows the participant flow from the number of families assessed for eligibility through to analysis. Of those reached, 6.4% (i.e. 28 families) expressed interest; initial interest came from 23 mothers and 5 fathers. Expressions of interest occurred at a rate of 3–4 families/week or 5–6 families/school assembly conducted. Less than half of those families who expressed interest (*n* = 28 families) participated in FRESH (*n* = 12 families) and were enrolled at a rate of 1–2 families/week. All families were retained at follow-up.

Of the 12 families enrolled, 4 were whole families and 6 were dyads (i.e. one parent and one index child); 32 family members participated overall. About 2–3 family members took part per family (range = 2–4); 4 families had an eligible additional adult, 3 families had an eligible additional child, and 1 family had both. Table 3 describes the participant characteristics.

**Table 3:** Individual participant characteristics at baseline

	Adults (*n* = 18)	Children (*n* = 14)
Sex (% male)	38.9	50.0
Age, year (± SD)	39.8 ± 8.2	8.3 ± 1.7
Height, cm (± SD)	168.6 ± 8.6	133.6 ± 12.7
Weight, kg (± SD)	74.7 ± 15.9	32.5 ± 10.8
Body mass index, kg m^−2^ (± SD)	26.3 ± 5.8	N/A
Body mass index *Z*-score (± SD)	N/A	0.5 ± 1.1
Waist circumference, cm (± SD)	92.0 ± 12.7	66.6 ± 12.3
Blood pressure, mmHg (± SD)		
Systolic	127.8 ± 16.2	110.0 ± 105
Diastolic	72.6 ± 9.1	64.6 ± 7.2
Pulse rate	68.3 ± 7.1	81.0 ± 7.8

After asking families about perceived challenges, focus groups revealed 4 challenges to be considered for optimising future recruitment. A brief description of the challenges is provided below, with supporting quotes in Table 4 (a–d).

**Table 4:** Supporting quotes from family focus groups

Findings related to recruitment and retention	
Sub-heading	Supporting quotes
a) Children trying to convey what FRESH was to parents	*“*I guess because you did it in assemblies, I wasn’t sure what he was going on about. It wasn’t till [J – a mother of a participating family] had obviously been in touch with you that we found out more. But going back to the whole thing of trying to then explain [FRESH], if the kids cannot do it, it becomes sort of Chinese whispers between the parents, does not it?” [Mother 7, family arm].
b) Participation would be time consuming	*“*I think people have misconceptions… I think they just heard the words research project and thought, ‘oh no, we are going to have to do a load of stuff’… but you do not have to do anything, just wearing this [pedometer] and going about what I do normally and log on the website every night or a couple of times a week and have a look at how we are doing. I did not think it was a hassle at all.” [Father 5, child-only arm].
c) Lack of confidence for physical activity	“Exercise is a funny thing, you know... Like if they are overweight or they do not eat healthy… they may think they are being judged by it and actually they are not being judged by it at all. That’s not what this was about... but there’s a fear factor when it comes to exercise for some people…. And given that I think obesity levels are pretty high around here for the national average, I think West Norfolk’s one of the fatter areas, people may be a bit... I do not know, possibly there was lack of confidence about signing up to something like this.” [Mother 5, child-only arm].
d) Reluctance to being measured	*“*It was the measurements, I would’ve done the other stuff… I think with some people that just puts you off straightaway. I think it did for me… I was like ‘no, I do not want to do that’ and I am sure others felt the same. Luckily [I - Father] did not mind because she really wanted to do this.” [Mother 12, family arm].
Findings related to intervention feasibility, acceptability, fidelity, and optimisation	
Sub-heading	Supporting quotes
e) Feasibility and acceptability of FRESH	*“*Definitely more aware, I underlined that [on the process evaluation questionnaire] because I think in terms of our awareness, it has made us a lot more aware of the steps that we are doing. I really, really liked that, for me that has been the best thing.” [Father 6, family arm].
	“…you [speaking about index child] wanted to walk more did not you, like if we were going to nursery you were like, ‘can I walk because I want to get more steps.’ I noticed that on a few things, whereas before she would have been like, ‘oh, can we go in the car?’” [Mother 12, family arm].
	“I do think if you’d given step counters to everyone in the family it gives us more onus to do it. Once you’d gone, it was all about him and no one else in the family, I felt like I’d done my bit and it was all down to just him and his step counter; whereas, if I’d have had a step counter… for the 6 weeks I probably would have been more aware about how active I was, and not necessarily competed with you, but just the fact that I had my own one to keep an eye on how active I’d been, then I’d have probably felt more involved.” [Father 8, child-only arm].
f) Family time	“We would actually compare on a daily basis… we’d be like ‘who’s done the most steps today?’ and you know, ‘oh, you have done more than you normally do, [B – index child]’ or ‘you have done less then you normally do.’ So, we were able gauge, ‘oh, it’s been a slow day, why has been it slow day? What have you been doing at school today?” [Father 6, family arm].
	“We had the planner out the whole time in the kitchen, so it was easier to fill in. [B – index child] was involved with it because, at the end of the day, I would say,’ have you written your log?’ And before bed she would have a look and she would write her number down and [Father] and I would shout our numbers to her and say, ‘oh this is mine, put mine in.’” [Mother 6, family arm].
g) FRESH website	“We pretty much just went on [the website] to log [steps]… I think we found that hardest thing, we would fall out over whose going to log [on the website]… so that wasn’t that helpful for the family dynamic (laughs).” [Father 6, family arm].
	“Well I’d like to have a leaderboard, that shows everyone doing it and it says, ‘you have got to beat this person and their name’, like it says on my football game.” [Boy 5, child-only arm]. “Yeah, a family one would be good. That would spur us all on would it not! It would spur us all on massively, yeah.” [Father 5, child-only arm].
h) Rewards	“He enjoyed that [virtual badges], but… maybe do a certificate or stickers or something, you know, even if you posted one to them, so they receive the post and we could be like ‘oh yeah, look what you have done!’ and… especially if you named it to them personally, so they actually got the physical post… ‘I have got a letter, I get to open that, wow, got my certificate in it!” [Mother 3, family arm].

#### Children trying to convey what FRESH was to parents

Delivering school assemblies emerged as an effective strategy for captivating children’s interest in FRESH, so much so that it appeared to be the main reason parents expressed interested in participating. However, children struggled, or were unable, to explain what FRESH was to their parents, likely impacting on the recruitment of the family unit.

#### Participation would be time consuming

Parents suggested that one of the main barriers was the perception that participation in FRESH would be burdensome and time consuming. However, participating parents reported that FRESH participation did not impede upon their normal daily activities.

#### Lack of confidence for physical activity

One family suggested a major challenge in recruiting families in their county might be due to a high prevalence of obesity, where they suggested families would be reluctant to register for a physical activity intervention due to a lack of confidence.

#### Reluctance to being measured

It was also confirmed that some family members chose not to participate in FRESH because they did want to participate in measurement sessions.

Families also suggested strategies, via focus groups, for improved recruitment, which included the following: a return visit to schools to give parents an opportunity to hear about FRESH and ask questions; exploring recruitment strategies that targeted adults through formal (e.g. employers) or informal settings (e.g. clubs, local fetes, shopping centres); using social media, such as Facebook or Twitter; and providing endorsements from previous participants or familiar organisations.

### Findings related to intervention feasibility, acceptability, fidelity, and optimisation

#### Feasibility and acceptability of FRESH

All children reported that they liked taking part in FRESH and thought it was fun. Table 5 (a) shows adults’ overall perceptions of FRESH. Scores were generally positive. In particular, adults agreed that FRESH was fun, encouraged their family to do more physical activity, and made their family more aware of the amount of physical activity they do, which was confirmed in focus groups (see Table 4e). Goal-setting also emerged as a major theme, particularly in those randomised to the family arm. Participants (adults and children) were aware of their required daily step counts to complete their weekly challenge and were able to identify ways to accumulate additional steps to meet daily targets (e.g. active travel; see Table 4e). Participants also reported receiving socio-emotional (e.g. feeling ‘closer’ as a family) and perceived cognitive benefits (e.g. in index child’s maths ability) through their participation. Lastly, all 6 families allocated into the child-only arm demonstrated a clear preference to have their whole family involved in FRESH. This finding was particularly evident among fathers (see Table 4e).

**Table 5:** Summary process evaluation findings for adult participants assessing the acceptability of the Families Reporting Every Step to Health (FRESH) intervention

	Overall	Family arm (*n* = 8 adults)	Child-only arm (*n* = 6 adults)
a) The FRESH study…			
…was fun for my family and I.	4.2 ± 0.8	4.3 ± 0.7	4.2 ± 1.0
…encouraged my family and I to do more physical activity.	3.9 ± 0.8	4.0 ± 0.6	3.8 ± 1.0
…has led my family and I to do more physical activity than we did before FRESH.	3.5 ± 1.0	3.6 ± 0.7	3.2 ± 1.3
…has led my family and I to do more activities (other than physical activity) together than we did before FRESH.	3.4 ± 0.7	3.3 ± 0.7	3.5 ± 0.8
…has made my family and I more aware of the amount of physical activity we do.	4.6 ± 0.5	4.7 ± 0.5	4.6 ± 0.5
…is something my family and I would like to continue to be part of.	3.8 ± 1.3	4.3 ± 0.8	3.4 ± 1.5
b) Regarding family time, to what extent do you agree or disagree with the following:			
It was easy to schedule ‘family time’.	3.1 ± 1.1	3.1 ± 1.4	3.1 ± 0.8
My family consistently scheduled ‘family time’.	3.1 ± 1.2	3.1 ± 1.4	3.2 ± 1.0
My child reminded us about ‘family time’.	2.9 ± 1.6	3.0 ± 1.7	2.7 ± 1.5
My child led/initiated ‘family time’.	2.6 ± 1.4	2.7 ± 1.5	3.0 ± 1.5
c) Regarding the FRESH website, to what extent do you agree or disagree with the following:			
It was easy to use.	3.6 ± 1.3	3.6 ± 1.4	3.5 ± 1.4
I enjoyed using it.	3.6 ± 0.9	3.6 ± 1.1	3.7 ± 0.8
My child/children enjoyed using it.	4.0 ± 1.2	3.9 ± 1.3	4.2 ± 1.0
I thought the website was appealing.	3.7 ± 0.9	3.6 ± 0.8	3.8 ± 1.0
I liked that there were varying degrees of difficulty with the challenges.	4.3 ± 0.9	4.3 ± 1.1	4.3 ± 0.8
I enjoyed the information about the cities.	3.9 ± 1.1	3.6 ± 1.3	4.3 ± 0.8
My child/children enjoyed the information about the cities.	3.8 ± 1.1	3.4 ± 1.3	4.3 ± 0.8
The step converter was useful (e.g., converting swimming to steps).	3.3 ± 1.3	2.8 ± 1.2	4.0 ± 1.2
The resources page was useful.	3.2 ± 1.1	3.0 ± 1.2	3.5 ± 1.0
I enjoyed the recipes.	3.3 ± 0.9	3.1 ± 1.1	3.5 ± 0.8
My child/children enjoyed the recipes.	3.4 ± 1.0	3.1 ± 1.1	3.7 ± 1.0
Logging our steps was easy.	3.7 ± 1.5	3.9 ± 1.7	3.5 ± 1.4
d) Regarding the step counter we gave out to log your steps, to what extent do you agree or disagree with the following:			
I did not mind wearing it.	4.0 ± 1.2	4.0 ± 1.2	N/A
My child/children did not mind wearing it.	3.9 ± 0.9	3.9 ± 1.1	4.0 ± 0.6
It was easy to use.	4.5 ± 0.7	4.6 ± 0.5	4.3 ± 0.8
I thought it was reasonably reliable at counting steps.	4.3 ± 0.7	4.6 ± 0.5	3.8 ± 0.8
I used the memory feature to go back and look at the number steps my family and/or I took.	4.6 ± 0.5	4.6 ± 0.5	4.5 ± 0.5

Participants responded on a 5-point Likert scale for each question (1 = strongly disagree; 5 = strongly agree)

#### Intervention fidelity

Using a 5-point Likert-scale (1 = strongly disagree, 5 = strongly agree), all families felt the kick-off meeting was useful (family vs child-only: 4.4 ± 0.8; 4.5 ± 0.8) and appreciated that it was a face-to-face meeting as opposed to a phone or video meeting. Most families felt they had enough technical support (3.9 ± 1.5, 4.2 ± 1.0), and the majority of families stated that a single meeting was enough for them to understand the protocol and how to use intervention website and materials. However, two families would have liked a follow-up meeting the following week.

##### Family time

Overall, adults disagreed that children led or reminded them of family time (see Table 5b). In line with the adult data, the majority of children did not perceive themselves to be their family’s team captain to lead on family time. Several children cited that they forgot they were team captain or they could not be bothered to be the team captain. There was also evidence to suggest that some parents took over the team captain role.

Overall, adults reported that it was not particularly easy for their family to schedule family time or to have it consistently. Most families claimed they either rarely or never had family time. A lack of time was the most commonly cited challenge for not having family time. Also, for some, parents’ work schedule (i.e. shift work) made it difficult to organise family time with all family members present. However, focus group evidence shows that some families were having discussions about physical activity in a manner that would be unlikely prior to FRESH (see Table 4f).

Generally, families only used their action planners to log daily step counts and not to plan weekly activities or anticipate barriers to meeting step goals. Most families preferred writing their step counts out on their paper-based action planners and transferring them onto the FRESH website once, near the end of their weekly challenge (see Table 4f).

##### FRESH website

Compared to the child-only arm, the family arm exhibited greater website engagement as they travelled to more cities (36 ± 11 vs. 13 ± 8) and failed fewer challenges (1.5 ± 1 vs. 3 ± 1). All children in the family arm and most (~ 80%) in the child-only arm wanted to continue using the FRESH website. Children in the family arm also found it easier to use the website, compared to those in the child-only arm (83% vs. 60%). Overall, adults’ mean scores were generally positive in relation to the FRESH website (see Table 5c), although more critical opinions were voiced during the focus groups. For the majority of families, the extent of their website engagement entailed selecting challenges and logging steps, which was normally a task performed reluctantly by parents (see Table 4g). Many adults and children were unaware or had not used several of the website elements (e.g. step calculator, parent resources, virtual rewards). Others stated that children were interested in the website (e.g. information about cities), but that interest wore off and only an interest in accumulating steps remained.

Website technical issues arose, particularly with the algorithm that calculated the number of steps families needed to accumulate to complete their challenge. This may have negatively affected some participants’ experience. Aside from technical bugs that needed resolving, families provided input on other potential improvements that could be made to the website. Almost unanimously, families wanted an element of competition on the website. It was evident from numerous focus groups that within-family competition occurred throughout the duration of the intervention period. However, the ability to compete against other families was also suggested in several focus groups (see Table 4g). Other suggested website improvement included: (1) adding a step history page to enable families to view progression over the intervention period, (2) more feedback/praise from the research team, (3) more flexibility in challenge destinations, (4) sending a text or e-mail reminder to log steps, and (5) an improved website design.

##### Pedometers

Overall acceptability of the pedometers was high for adults in both arms (Table 5d). Generally, adults stated that it became a ‘routine’ or ‘second nature’ to wear pedometers, although some would have preferred wrist-worn pedometers. The most frequently cited reason children gave for wanting to participate in FRESH was to receive a pedometer. Families reported that there were few settings where children were not allowed to wear their pedometers, with the most cited setting being during physical education. Pedometer wear was more acceptable to children in the family arm than the child-only arm (~ 80% vs 60%).

##### Rewards

Overall, parents moderately agreed that their child enjoyed receiving virtual rewards (3.5 ± 1.2), with slightly higher scores in the child-only arm compared to the family arm (3.8 ± 1.0 vs. 3.1 ± 1.3). Children’s focus group responses generally supported parents’ perceptions that the virtual rewards were not particularly of long-term interest to them. Most parents suggested a small tangible reward would appeal to their child more than a virtual reward, such as posted certificates or stickers. Other suggestions included vouchers, clothing, or equipment that encouraged physical activity (see Table 4h).

##### Risk of contamination

Focus groups revealed that children were aware of other FRESH participants in their school and that some families did indeed communicate among each other about FRESH, with some even revealing their allocated condition. We also discovered that a family allocated to the child-only arm purchased a set of pedometers for their family.

### Findings related to feasibility of outcome evaluation

Data collection took an average of 91.1 ± 27.7 min/family at baseline and 77.1 ± 24.5 min/family at follow-up. Overall, adults disagreed that there were too many measures and that data collection took too long and all children self-reported that they ‘liked’ being measured. With the exception of accelerometer/GPS and step test assessment (1 refusal each), all participants completed all measures at baseline. At follow-up, 91% of participants accepted an accelerometer/GPS and completed the step test; 94% of participants completed all other measures.

At baseline, valid accelerometer wear was 851.5 ± 54.1 and 755.7 ± 29.7 min for adults and children, respectively, and 843.1 ± 78.6 and 742.3 ± 56.4 at follow-up, and the GPS provided a location for 750.6 ± 191.4 and 646.2 ± 189.0 min at baseline and 720.0 ± 237.6 and 586.8 ± 262.8 at follow-up. Valid data on ≥ 4 days (including 1 weekend day) was available for 83% of adults at baseline and follow-up; this was slightly lower for children, at 75% and 67%. Visual inspection of wear time data revealed a tendency for children to remove their devices around dinner time, parents to remove their devices after their child went to bed, and families to put on their devices much later in the day at the weekend compared with weekdays.

Initial assessment of family functioning via the video-recorded Fictional Family Holiday activity showed poor-to-moderate data quality as discussions were limited and cursory. Three factors may have affected data quality: (1) most families enrolled were dyads, limiting opportunities for whole-family discussion; (2) providing families with a planner to write out their itinerary may have shifted the emphasis away from open-ended discussion; and (3) the activity was completed at the end of the visit, when participants may have been fatigued from data collection.

The physical activity-related expenditure questionnaire developed for this study appeared to have appropriate face validity and was capable of providing rich data related to membership fees and subscriptions (e.g. for sports clubs, fitness centres, after school clubs) and sports equipment (e.g. sportswear, gadgets).

## Discussion

The current study provides a response to calls for the need for innovative interventions targeting young people and families [18]. To our knowledge, FRESH is among the first physical activity interventions to specifically target whole family engagement, helping to create supportive, synergistic environments for the promotion of healthy behaviours and long-term change [11, 17, 27]. Here, we assessed the feasibility and acceptability of FRESH to inform future research. Our findings showed that it was feasible and acceptable to deliver and evaluate a family-targeted physical activity promotion intervention with generally high acceptability from participating families. This feasibility study, however, also revealed areas for improvement.

### Optimising recruitment

Previous literature has identified family-based recruitment as being particularly difficult [14, 56]. Our formative work [26] and other studies (see a review by Morgan et al. [25]) recommend a multi-faceted recruitment strategy in family-based research. Due to unforeseen delays, we were unable to employ our planned multi-faceted recruitment strategy, which likely contributed to our under-recruitment of families (60% of targeted 20). Of the families enrolled, only 1/3 included all family members. There was some suggestion that this may have been due to a lack of confidence for physical activity or a reluctance to be measured. Improved messaging is therefore required early in the recruitment process to reassure low-active families that FRESH is tailored to their activity levels and highlight the option of opting out of (parts of) the measurements. Allowing family members to be involved in the intervention, regardless of their participation in the evaluation, may improve effectiveness and long-term behaviour change [14–17].

Interestingly, our findings showed that fathers appeared to be interested in participating in FRESH but only 5 out of 28 expressions of interest were initiated by fathers. This may be because, among heterosexual parents, tasks such as making phone calls (e.g. to express interest) or family event preparation (e.g. study participation) are more likely to be performed by mothers than fathers [57]. Therefore, recruiting whole families, where any parent could initiate an expression of interest, may be an important catalyst for the inclusion of more fathers in family-based research.

Other key areas of improvement to recruitment include the following: optimising the conversion from children reached to expressions of interest (e.g. extending the age range of index children to cover the whole of key stage 2; reducing the burden on children to explain FRESH, instead directing parents to a video); targeting adults via community- and employer-based recruitment or social media; and obtaining recruitment support from local organisations.

### Optimising the FRESH intervention

FRESH is designed as a goal-setting and self-monitoring intervention aimed at increasing family physical activity. Encouragingly, these behaviour change techniques resonated with most families and align with recommendations to increase family physical activity [27]. Participants reported being aware of what their daily step goals needed to be in order to complete their weekly challenges. Interestingly, the challenge context did not seem to be important to participating families (i.e. choosing challenge cities to walk to virtually). Instead, focus group interviews revealed that meeting daily step goals, completing weekly challenges, and intra-family competition appeared to be key drivers motivating families throughout the intervention period.

We found that families were not implementing all intervention components as intended and strategies to improve intervention delivery and families’ fidelity to the intervention protocol may be needed. For instance, most families were not selecting new challenges on the FRESH website together during family time, and families were only using their action planners to log their steps, not to also identify family activities or upcoming challenges for the week ahead. During the kick-off meeting, the facilitator could place a greater emphasis on family time and help the family schedule it. Facilitators are critical to the delivery of interventions and a recent review found that facilitators have an important moderating influence on the effectiveness of any programme [58]. Ongoing follow-up with the facilitator would also support this. Other strategies to improve intervention fidelity include e-mail reminders to log steps, adding competition elements to the website (e.g. a leaderboard), more regular feedback/praise from the research team, and including small tangible rewards.

Although the FRESH intervention overall was well-received, in our focus groups, it was evident that families and, in particular, fathers in the child-only arm expressed that they would have preferred having their whole family involved in FRESH. Discontinuing this study arm should therefore be considered. Further, the finding that fathers were particularly interested in having their whole family participate in FRESH is noteworthy. Fathers have an independent influence on their children’s health and development [59] and an important influence on children’s physical activity [60–62], but they are grossly underrepresented in family-based interventions [63]. Fathers’ engagement with FRESH is consistent with recent evidence that fathers are more willing to participate in family-based interventions when the focus is on their children [64] and, as a result, reported newfound enjoyment for family-based physical activity and a desire to be a positive role model [65]. The online delivery of FRESH may have also appealed some fathers [64].

### Optimising measurement

The duration of data collection at both time points was in line with our estimates and acceptability of the duration, and number of measures was high for both adults and children. Nevertheless, it may have acted as a barrier to participation. Minor adjustments are needed to improve the quality of the expenditure and family functioning data and monitor wear time. For example, a greater emphasis on recruiting whole families, removal of the written aspect of the activity, and shifting the order of measures, so that the Fictional Family Holiday activity occurs earlier during data collection, might improve the quality of the family functioning data. To improve wear time, emphasis should be placed that each individual participant should wear the monitors for as long as possible from the time *they* wake up until the time *they* go to sleep as opposed to children’s bedtime. Also, reminders (e.g. e-mail, phone) could improve wear time [66], particularly at the end of the week to improve weekend wear.

### Strengths and limitations

This study is among the first physical activity interventions that aimed to target and measure whole families, providing novel evidence in an area where more primary research is needed [18]. The phased approach of assessing feasibility and acceptability to inform refinement for pilot study is in accordance with established guidelines [67]. Public involvement was used extensively to inform development and refinement of FRESH, as suggested previously [68, 69]. Further, our use of a mixed-methods design provides unique insight and context for our quantitative findings, assisting in identifying strategies to further optimise FRESH. Limitations include that we were unable to fully employ our recruitment strategy and did not have the opportunity to test the efficacy of recruiting families through community-based recruitment. Additionally, only one-third of families enrolled in FRESH included all family members.

## Conclusion

In conclusion, this study demonstrates feasibility and acceptability of the family-targeted FRESH intervention and provides valuable suggestions for further optimisation. This work informs a future pilot trial testing the impact of these adaptations and the preliminary effectiveness of FRESH on family physical activity. The findings of our upcoming pilot trial will inform sample size/power calculations for a future definitive trial, should the pilot study findings suggest a definitive trial is warranted.

## Abbreviations

FRESH: Families Reporting Every Step to Health
GPS: Global positioning system
MVPA: Moderate-to-vigorous physical activity
